# HEART Score in Predicting One-Month Major Adverse Cardiac Events in Patients with Acute Chest Pain; a Diagnostic Accuracy Study

**DOI:** 10.22037/aaem.v9i1.1052

**Published:** 2021-03-27

**Authors:** Hossein Alimohammadi, Majid Shojaee, Mohammad Reza Sohrabi, Saman Salahi

**Affiliations:** 1 Emergency Department, Imam Hossein Hospital, Faculty of Medicine, Shahid Beheshti University of Medical Sciences, Tehran, Iran.; 2 Faculty of Medicine, Shahid Beheshti University of Medical Sciences, Tehran, Iran. Email:

**Keywords:** Chest pain, heart diseases, Heart Disease Risk Factors, Acute Coronary Syndrome, Emergency Service, Hospital

## Abstract

**Introduction:**

Screening of high-risk patients and accelerating their therapeutic procedures can reduce the burden of acute coronary syndrome (ACS). This study aimed to evaluate the accuracy of HEART score in predicting the risk of one-month major adverse cardiac events (MACE) in these patients.

**Methods:**

In this prospective cross-sectional study, the accuracy of HEART score in patients over 18 years old who presented to emergency department following acute chest pain, was evaluated during a 21-month period. Each patient was followed up regarding the incidence of MACE for one month via phone call and the hospital’s integrated health information system.

**Results:**

240 cases with the mean age of 60.50 ± 16.07 years were studied (56.3% male). MACE was observed in 77 (32.1%) cases. The most common MACE was percutaneous coronary artery revascularization (PCAR) (12.9%). The mean HEART score of studied cases was 4.74 ± 2.12. The mean score of cases with MACE was significantly higher than others (6.25 ± 1.97 versus 4.03 ± 1.79; p < 0.0001). Based on this score, the risk of MACE was high in 34 (14.2%), moderate in 118 (49.2%), and low in 88 (36.7%) cases. The incidence of one-month MACE was 85.3% in high-risk cases, 35.6% in moderate one, and 6.8% in low-risk cases based on HEART score. The area under the ROC curve of HEART score in predicting the risk of MACE was 0.796 (95% CI: 0.736 – 0.856). The best cut off point of HEART score in this regard was calculated as 4.5. The sensitivity and specificity of this score in 4.5 cut off were 83.11% (95% CI: 72.49 – 90.35) and 66.25% (95% CI: 58.38 – 73.35), respectively.

**Conclusion:**

Based on the findings of the present study the mean HEART score of ACS patients with one-month MACE was significantly higher than others and the incidence of MACE in high-risk patients was significantly higher. But the overall accuracy of score in predicting one-month MACE in ACS patients was in moderate range.

## Introduction

Cardiovascular diseases are currently the most common cause of morbidity and mortality among men and women of all races and ages worldwide, the most prevalent of which is Coronary Artery Disease (CAD) (1-3). According to the American Heart Association, these diseases are the cause of one in three deaths in the United States and an average of about 2,200 Americans die from these diseases each day (4). Based on research done on this matter, the rate of heart disease in Iran has increased by 20 to 25 percent in recent years (5). Human error is considered a major risk factor in diagnosing these accidents (6) and preventing these errors is one of the most important and common challenges in the healthcare system (7). Also, chest pain is one of the most common causes of emergency room visits, which may be due to life-threatening conditions such as serious coronary artery disease (8). This disease is one of the most prevalent, costly, and debilitating diseases. Hence, identifying the factors that can help in reaching a rapid diagnosis is of great importance (9). Correct and rapid detection of serious coronary artery disease is crucial as undiagnosed cases had a high mortality rate and constituted the majority of medical malpractice lawsuits in the United States in 2016 (10). The HEART score is a simple scoring tool for determining the risk of ischemic events in patients with acute chest pain, who presented to the emergency department, which is designed to differentiate the group of patients who can be quickly discharged from the emergency department (11) from those who need to be hospitalized for further investigations or treatments. Rapid, accurate, and reliable results enable the physician to discharge low-risk patients with high confidence and without additional tests, and to quickly identify high-risk patients for future invasive measurements and reduce the probability of error (12, 13). Considering that evaluation and monitoring of patients referring to the hospital with chest pain is very important and costly, scoring systems such as HEART are designed to prevent unnecessary hospitalization of patients and reduce inpatient expenses at the time of admission, which results in reduced stress and anxiety as well. The present study aimed to evaluate the accuracy of HEART score in predicting the risk of one-month major adverse cardiac events (MACE) in patients presenting to emergency department following acute chest pain.

## Methods

***Study design and setting***

In the present cross-sectional prospective study, the accuracy of HEART score in predicting the risk of one-month MACE in patients who presented to the emergency department (ED) of Imam Hossein Hospital in Tehran, Iran, from May 2018 to March 2020, complaining of acute chest pain, was assessed. The researchers were committed to following the ethical principles of clinical research and all the patient data were kept confidential. Methodology of the study was approved by the Ethics Committee of Shahid Beheshti University of Medical Sciences (ethics code: IR.SBMU.MSP.REC.1397.250).

***Participants***

The required sample size for this study was calculated to be 225 cases, using the Cochran formula. Of the 406 patients over 18 years of age who arrived at the emergency department of Imam Hossein hospital in Tehran complaining of chest pain during the above-mentioned time period, 240 cases who had sufficient follow-up data were entered in the study via census sampling method. Cases with incomplete medical profile, those who died due to any non-cardiac reason, and patients who refused to cooperate, were excluded.

***Data gathering***

Demographic data (age, sex), history of present illness, electrocardiographic (ECG) findings, risk factors of coronary artery disease (hypertension, hypercholesterolemia, diabetes mellitus, previous cardiac disease, smoking, obesity, and atherosclerosis), and initial serum Troponin level, which are all noted in HEART score, were gathered using a predesigned checklist. This information was collected by a general physician or an emergency medicine resident at the time of the patient’s first visit to the ED under the direct supervision of emergency medicine specialists. MACE was defined as: acute myocardial infarction (AMI), death due to acute cardiac event, coronary artery bypass graft (CABG) surgery, percutaneous coronary artery revascularization (PCAR). These data were extracted and gathered by a senior emergency medicine resident through phone calls and reviewing the hospital Health Information System (HIS).

***Calculating patients’ scores***

In this scoring scale, HEART is an abbreviation, which stands for History, ECG, Age, Risk factors, and Troponin level; each one gets a score of 0, 1, or 2 (14). Total scores between 0-3 define low-risk cases, scores 4-6 indicate moderate risk, and scores 7-10 are indicative of high-risk cases. 

***Statistical analysis***

Convenience sampling was used for this study. After entering data to a designed excel sheet, they were analyzed using SPSS 21 statistical software. To report the findings, frequency and percentage, or the descriptive-mean statistics were used. Chi-square and t-test were also used for analysis of data. The area under the receiver operating characteristic (ROC) curve of HEART score was used for calculating the accuracy and the best cut-off point of this score in predicting one-month MACE. Screening performance characteristics of HEART score at the best cut-off point was calculated and reported with 95% confidence interval (CI). Level of significance was considered to be 0.05.

## Results

***Baseline characteristics of cases***

Throughout the 22 months that this study was performed, a total of 406 patients presented to the emergency department complaining of acute chest pain and were evaluated. 166 (40.8%) cases were excluded from the study due to missing data or their unwillingness to participate. The remaining 240 qualified cases were entered to the study (56.3% male). The mean age of patients was 60.50 ± 16.07 (22 – 95) years. Table 1 shows the baseline characteristics of studied cases. MACE was observed in 77 (32.1%) cases. The most common MACE was PCAR (12.9%). 

***Accuracy of HEART score***

The mean HEART score of studied cases was 4.74 ± 2.12. The mean score of cases with MACE was significantly higher than others (6.25 ± 1.97 versus 4.03 ± 1.79; p < 0.0001). Based on this score, the risk of MACE was high in 34 (14.2%), moderate in 118 (49.2%), and low in 88 (36.7%) cases. There was a significant correlation between the HEART score's predicted risk of MACE and experience of MACE (p < 0.0001). The incidence of one-month MACE was 85.3% in high-risk cases, 35.6% in moderate ones, and 6.8% in low-risk cases based on HEART score.

**Figure 1:**
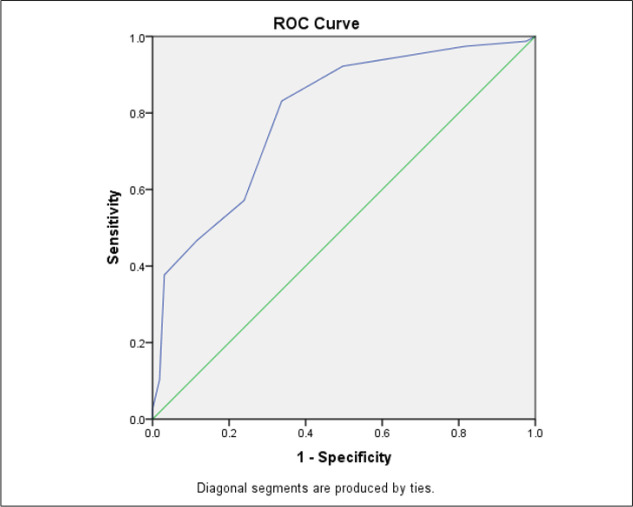
Area under the receiver operating characteristics (ROC) curve of HEART score in predicting major adverse cardiac events in patients with acute coronary syndrome

The area under the ROC curve of HEART score in predicting the risk of MACE was 0.796 (95% CI: 0.736 – 0.856). The best cut-off point for the score in this regard was calculated as 4.5 (figure 1). The screening performance characteristics of this score in 4.5 cut-off point is presented in table 2. The sensitivity and specificity of the score in 4.5 cut-off were 83.11% (95% CI: 72.49 – 90.35) and 66.25% (95% CI: 58.38 – 73.35), respectively.

## Discussion

Based on the findings of the present study the mean HEART score of ACS patients with one-month MACE was significantly higher that others and the incidence of MACE in high-risk patients was significantly higher. But the overall accuracy of this score in predicting one-month MACE in ACS patients was in moderate range with 83.11% sensitivity and 66.25% specificity.

HEART score is a tool for facilitating making diagnostic and therapeutic decisions regarding patients with chest pain who present to the emergency department, without the use of radiation or invasive methods. It seems to be an easy, rapid, and reliable predictive tool for evaluating patients with chest pain, which can be used to triage these patients in the ED and also prevent further hospitalization for costly and time-consuming diagnostic work-ups in suspected but low-risk patients.

The results of the present study showed that the majority of patients with chest pain were categorized in the moderate-risk group, which is consistent with the study by Six et al. (15), while the study by Melki et al. stated that 60.2% of patients were categorized as a low-risk group (16); and also the study by Gharaee et al., which stated that the majority of patients with chest pain were in the low-risk group (17). 

The average HEART score was 4.03 in the group without MACE, and 6.25 in the group of patients with MACE, which supports the study by Backus et al. that reported the average scores of 3.96 and 6.54 in the group with no cardiac events and the group with MACE, respectively (18). 

The most common MACE in patients included percutaneous coronary artery revascularization, myocardial infarction, coronary artery bypass graft surgery, and death due to acute cardiac complications, respectively; which is consistent with the study by Backus et al. in 2013 (18). More than half of all patients who referred with chest pain showed no severe cardiac complication after a month, which is consistent with the results of Backus et al., indicating that 83% of patients showed no cardiac complications (18). 

High-risk patients based on the HEART score had the highest incidence of adverse cardiac events, which was consistent with the study by Backus et al. (18) in 2013, which might show the ability of HEART score to correctly categorize patients and predict their short-term outcome. 

The present study indicates that the incidence of MACE is 6.8% in the low-risk group of patients, while Backus et al. in a study titled “a prospective validation of the HEART score for chest pain patients at the emergency department” reached the result of 1.7% in low-risk group (18); and in a study by Mahler et al., this rate was reported to be less than 1% (14). The results of this study also indicate 85.3% incidence of adverse cardiac events in the high-risk group, which supports the studies by Leite et al. (19) and Melki et al., which reported that more than half of high-risk patients had adverse cardiac events (16). This is also consistent with the study by Backus et al., which reports 50.1% MACE in the high-risk group of patients (18). 

Data analysis of the present study showed that there is a statistically significant relationship between HEART score variables and the incidence of MACE, which corresponds to the study by Mahler et al., stating that the incidence of adverse cardiac events is strongly correlated with HEART score (14). 

HEART score has recently been proposed as a tool for classifying patients with chest pain in high, moderate, and low-risk groups. But based on the findings of this study, it seems that using this tool should be considered with caution and as an adjuvant tool along with overall judgments of in charge physicians. 

**Table 1:** Baseline characteristics of studied cases

**Variables**	**Number (%)**
**Sex**	
Male	135 (56.3)
Female	105 (43.8)
**Age (years)**	
<45	42 (17.5)
45-65	110 (45.8)
≥65	88 (36.7)
**Suspicion for ACS**	
Low	12 (5.0)
Moderate	80 (33.3)
High	148 (61.7)
**Electrocardiography findings**	
Normal	121 (50.4)
Non-specific repolarization disturbance	75 (31.3)
Significant ST depression	44 (18.3)
Number of risk factors*	
0	79 (32.9)
1-2	108 (45.0)
≥3	53 (22.1)
**Initial troponin level (ng/ml)**	
< 0.05	158 (65.8)
0.05 - 0.12	62 (25.8)
> 0.12	19 (7.9)
**Adverse cardiac events**	
Percutaneous coronary artery revascularization	31 (12.9)
Acute myocardial infarction	26 (10.8)
Coronary artery bypass graft surgery	9 (3.8)
Death due to acute cardiac complication	11 (4.6)
No acute cardiac complication	142 (59.2)

* Diabetes mellitus, smoking, hypertension, hypercholesterolemia, obesity, atherosclerosis, family history of coronary artery disease. ACS: acute coronary syndrome.

**Table 2:** Screening performance characteristics of HEART score in predicting major adverse cardiac event in patients with acute coronary syndrome at 4.5 cut-off point

**Character**	**Value (95% CI)**
Sensitivity	83.11 (72.49 – 90.35)
Specificity	66.25 (58.38 – 73.35)
Positive predictive value	53.78 (44.43 – 62.88)
Negative predictive value	89.25 (81.99 – 93.92)
Positive likelihood ratio	1.16 (.90 – 1.50)
Negative likelihood ratio	0.12 (0.07 – 0.20)

CI: confidence interval.

##  Limitations

Small sample size, short time period of follow-up, high number of excluded cases, and probability of selection bias might be among the most important limitations of the current study. Moreover, the selection of patients for the type of intervention needed to address the MACE was based on the in-charge physician’s decision and not exactly a determined standard, which may have caused errors in selection of patients.

##  Conclusion

Based on the findings of the present study the mean HEART score of ACS patients with one-month MACE was significantly higher that others and the incidence of MACE in high-risk patients was significantly higher. But the overall accuracy of this score in predicting one-month MACE in ACS patients was in moderate range with 83.11% sensitivity and 66.25% specificity.
